# The Gorilla: Being a Sketch of Its History, Anatomy, General Appearance and Habits

**Published:** 1861-12

**Authors:** 


THE GORILLA: Being a Sketch of its History, Anatomy, General Appearance and Habits. By LEONARD J. SANFORD, M. D., New Haven, Conn. Dec., 1861.

Our countryman, Du Chaillu, opened a rich vein of something more than nine days' wonder, when he returned from his "Adventures in Equatorial Africa," with the first entire skeletons and stuffed specimens of the largest authropoid ape yet seen. And though his reception here was anything but enthusiastic, British savans and the British public have certainly lionized him *ad nauseam*,—naturalists have feted him, curators and librarians have denounced him, and his proteges have been taken as far into the pulpit as the Rev. Mr. Spurgeon could conveniently get them.

Here, we are just beginning to get interested in the subject, and this pamphlet reprint,* by Dr. SANFORD, is the first monograph we have yet met with.

For fifteen years has this animal been known to modern naturalists, and yet with such an intertexture of fact and fiction, as to place him on the shadowy confines of romance, almost with the phœnix and the roc. The name itself, as Dr. SANFORD tells us, is much older, being used by the Carthaginian voyager, Hanno, B. C. 600, and was applied to the present species by Dr. Savage and Prof. Wyman, of the Soc. of Nat. Hist., at Boston, in 1847, to whom the Rev. J. L. Wilson, an African missionary, forwarded a skull and some

*From the *American Journal of Science and Arts*, Vol. xxxiii, Jan. 1862.

other bones of a skeleton from the Gaboon coast. Dr. S. gives a general sketch of the anatomy, comparing its striking points of resemblance with man, closing his remarks on the osseous system, as follows ;

The anthropoid apes take rank in relation to man, according to the degree of approach of their skeletons to his. By this criterion, the gorilla has a high, perhaps the highest position. His skull, as we have seen, has fewer human resemblances than those of some other species, but in the rest of his bony framework he stands much nearer the archetype. A comparison of the entire skeleton, among the series, leaves us in some doubt as to the exact place he should occupy. Professors Wyman and St. Hilaire put the chimpanzee first, and the gorilla second; while Prof. Owen states, that the tailless quadrumana recede from the human type, in the following order: viz, gorilla, chimpanzee, orang-œtan, gibbon.

*The muscular system* of the apes, throughout its entire structure and arrangement, conforms very closely to that of man. So also the structure and form of the *lungs* and *heart*, and the distribution of the *blood-vessels* and *nerves*, are all but identical with the corresponding organs in man. But the *brain*, though having the elliptic form of its human congener, differs from it considerably in size* and points of structure. In bulk, and in the number and size of its convolutions, the discrepancy is great. The *cerebellum*, relatively to the *cerebrum*, is larger than in man. This disparity of size, consequent upon the larger cerebellum, is a characteristic of the brute creation, and it increases up to a certain limit, as we recede from man in a descending series. It is indicative of excessive animalism, or rather of a preponderance of the purely animal functions.

The brain of anthropoid apes is distinguished from that of other brutes, in possessing a process of structure known as the *hippocampus minor*—this is a minute, nipple-shaped body, which is found in the posterior cornu or horn of each *lateral* (the largest cavities of the brain) *ventricle*. Its existence in the apes, to the exclusion of all other animals except man (for this, so far as known, is a fact), is the more remarkable, inasmuch as the posterior lobes of the brain, which contain the cornua, are very inconsiderably developed in them.

In the circle of their functions, and in the phenomena of

*The weight of brain in a full grown gorilla, is from 10 ounces to 12 ounces, troy; in the chimpanzee and kooloo-kamba, it is somewhat greater than this. In the full grown negro, it ranges from 3 pounds 1 ounce, to 3 pounds 9 ounces 4 drachms, troy.

periodicity, the apes, again, make a close approach to the human species.

The gorilla, in general configuration, is quite like the apes, but his larger size, and more compact organization, deprive him of their agility of motion—and so, by good right, of their name. In motion and manner, he must be an awkward and ungainly ape. He is accomplished, however, by possessing great strength—in this particular, as also in ferocity of disposition, we conceive him to be something terrible. His *physique* judged of by man's, is graceless and shabby in the extreme; in the comparison, we are justified in characterizing him, as Buffon has done the sloth, " a bungled composition of nature."

